# Insufficient classification of anaemia in general practice: a Danish register-based observational study

**DOI:** 10.1080/02813432.2021.1958499

**Published:** 2021-07-30

**Authors:** Astrid Boennelykke, Henry Jensen, Lene Sofie Granfeldt Østgård, Alina Zalounina Falborg, Kaj Sparle Christensen, Anette Tarp Hansen, Jon Emery, Peter Vedsted

**Affiliations:** a Research Unit for General Practice, Aarhus, Denmark; b Department of Public Health, Aarhus University, Aarhus, Denmark; c Department of Haematology, Odense University Hospital, Odense, Denmark; d Department of Clinical Epidemiology, Aarhus University Hospital, Aarhus, Denmark; e Department of Clinical Biochemistry, Aalborg University Hospital, Aalborg, Denmark; f Department of General Practice and Centre for Cancer Research, Victorian Comprehensive Cancer Centre, University of Melbourne, Melbourne, Australia

**Keywords:** General practice, anaemia, haemoglobins, primary health care, cross-sectional studies, Denmark

## Abstract

**Background:**

Anaemia can be a pointer of underlying severe disease, including undiagnosed malignancy. Subsequent blood tests are essential to classify the anaemia into subtypes and to facilitate targeted diagnostic investigation to ensure timely diagnosis of underlying disease.

**Objective:**

We aimed to describe and classify anaemia based on laboratory tests from patients with new-onset anaemia detected in general practice. An additional aim was to analyse associations between patient characteristics and unclassified anaemia (not classifiable according to an algorithm).

**Design:**

Population-based cross-sectional study.

**Setting:**

Danish general practice.

**Subjects:**

A total of 62,731 patients (age: 40–90 years) with new-onset anaemia were identified in Danish laboratory information systems and nationwide registries, and data were obtained for 2014–2018.

**Main outcome measures:**

We measured the proportion of patients classified into subtypes of anaemia based on blood tests requested by general practitioners within 31 days of the anaemia index date.

**Results:**

Of the 62,731 patients with new-onset anaemia, we identified unclassified anaemia in 78.9% (95% confidence interval (CI): 77.3–80.5) of men and 65.1% (CI: 63.4–66.9) of women. The likelihood of unclassified anaemia increased with age, increasing comorbidity and decreasing severity of anaemia.

**Conclusion:**

The majority of patients with new-onset anaemia could not be classified through a simple algorithm due to missing blood tests, which highlights a potential missed opportunity for diagnosis. Standardised laboratory testing of patients with anaemia is warranted to ensure adequate follow-up and early detection of underlying severe disease.

**KEY POINTS**
- Anaemia can be a sign of malignancy, and anaemia classification is an important step in the diagnosis of underlying disorders.
- The majority of patients with anaemia could not be classified according to a simple algorithm due to missing blood tests.
- Some patient characteristics were associated with a high risk of unclassified anaemia: high age, high comorbidity, and severe anaemia.
- Standardised laboratory testing in patients with anaemia is needed to inform targeted diagnostic investigation to ensure timely diagnosis.

## Introduction

Anaemia is associated with increased morbidity and all-cause mortality in the general population [1,2]. As even mild anaemia may have a negative impact on the prognosis, careful evaluations are essential [1,3]. Moreover, anaemia is an emerging health problem in the aging population, affecting 17% of the population aged 65+ years [4].

Anaemia is often an incidental finding in patients presenting with non-specific symptoms (e.g. fatigue and headache). It can be a sign of underlying disease, including undiagnosed malignancy [2,3]. Hence, all cases of anaemia should be investigated to reduce diagnostic delays in potentially serious and treatable diseases.

Haemoglobin measurement is a common laboratory test [5]. In Denmark, it can be requested as a single test without a full blood count. However, as the diagnostic workup in patients with anaemia largely depends on the type of anaemia, additional laboratory tests are needed to classify the anaemia and indicate relevant diagnostic investigations, e.g. endoscopy [6,7]. Hence, laboratory testing and anaemia classification is an important step in the diagnostic process in patients with anaemia. General practitioners (GPs) have a key role in the process of investigating and classifying anaemia, as they are often the first to detect anaemia. Few studies have evaluated laboratory findings in patients with anaemia, and existing studies were flawed by small sample size [8], including only an elderly population [9], or reporting no inclusion criteria for new-onset anaemia [8,9]. Furthermore, no previous studies have evaluated and classified patients with anaemia into different subtypes of anaemia based on the available blood samples. Likewise, no previous studies have investigated if an association exists between specific patient characteristics and unclassified anaemia.

We aimed to describe the classification of anaemia based on laboratory test results in patients with new-onset anaemia detected in general practice. Additionally, we aimed to analyse associations between patient characteristics and unclassified anaemia.

## Materials and methods

We conducted an observational population-based cross-sectional study using data from Danish laboratory information systems [10]. The data were linked with individual-level information from Danish nationwide registries through the unique civil personal registration (CPR) number [11,12].

### Setting

Denmark has a population of ∼5.7 million inhabitants and consists of five healthcare regions. This study is based on data from the Northern Denmark Region (0.6 million inhabitants) and the Central Denmark Region (1.3 million inhabitants) [13]. Citizens in Denmark have free access to healthcare services through the public tax-funded healthcare system [13]. Almost all citizens (99%) are registered with a general practice, which has a gatekeeping role and must be consulted for medical advice (except for emergencies, primary care otorhinolaryngologists, and ophthalmologists) [14].

All blood samples are analysed at a hospital-based clinical biochemistry laboratory and registered in the electronic laboratory information systems, except for point-of-care tests, which are analysed in general practice (e.g. haemoglobin and C-reactive protein, but they were not included in this study) [5,10]. The Danish civil registration (CPR) number is used to record all test results, including date of sample, reference interval, and requesting unit (GP or hospital department).

### Study population

All patients aged 40–90 years living in one of the two regions were eligible for inclusion if registered in the laboratory information system with new-onset anaemia based on a blood test requested by a GP in the period from 1 April 2014 until 30 November 2018. We excluded patients registered with anaemia in the laboratory information system (from general practice or hospital) in the 15 months preceding the date of anaemia registered in the inclusion period (index date). Re-entry into the cohort was not allowed. We excluded patients who moved in/out of the two regions in the study period and patients who died within 31 days from the index date.

### Exposure, outcome, and covariates

#### Exposure

The exposure was anaemia, which was defined as a haemoglobin level below 134 g/L for men and below 118 g/L for women according to the Danish reference intervals [15].

#### Outcome

The main outcome measures were aetiological [7,16] and morphological [17] subtypes of anaemia. To define aetiological subtypes of anaemia, we applied the diagnostic algorithm for unexplained anaemia by the Danish Society for Gastroenterology and Hepatology. We divided subtypes into four groups based on haemoglobin, ferritin and C-reactive protein (CRP): (i) iron deficiency anaemia (IDA): anaemia with ferritin <30 microgram/l (µg/l), (ii) combined inflammatory iron deficiency anaemia (CIIDA): anaemia with ferritin <100µg/l and increased CRP, (iii) anaemia of inflammation (AI): anaemia with ferritin >100µg/l and increased CRP, (iv) other causes: anaemia with ferritin >30µg/l and normal CRP[7,16]. If the anaemia could not be classified according to the algorithm due to missing blood tests, it was categorised as unclassified anaemia. The morphological subtypes were based on measures of erythrocyte morphology and classified according to the Danish reference intervals for mean cell volume (MCV) into three groups: microcytic [MCV <82 femtolitre (fL)], normocytic (MCV 82–98 fL), and macrocytic (MCV >98 fL) [17].

Subsequently, we studied measures of ferritin, C-reactive protein (CRP), red cell distribution width (RDW), cobalamin, folate, and repeated haemoglobin. We also studied the use of an anaemia ‘laboratory test package’ requested by GPs. This package is available in the Central Denmark Region, and it includes a full blood count as standard. The package is an active request from the general practitioner, and it triggers other relevant blood tests (based on measures of erythrocyte morphology) if anaemia is present (e.g. ferritin in hypochromic anaemia or cobalamin and folate in hyperchromic anaemia) [18]. Anaemia classification and laboratory tests were based on blood tests requested by GPs within 31 days of the anaemia index date. Repeated haemoglobin testing was defined as haemoglobin measurements requested by a GP within 12 months of the index date.

#### Covariates

Covariates in the analyses were sex, age, educational level, disposable income, civil status, anaemia severity, and comorbidities (number and type). Information on sex and age was retrieved from the Civil Registration System (CRS) [12]. Information on educational level, disposable income, and civil status was obtained from Statistics Denmark. Educational level was defined according to the International Standard Classification of Education (ISCED) and categorised into ‘low’, ‘medium’, and ‘high’. Disposable income was grouped into tertiles of ‘low’, ‘medium’, and ‘high’. Civil status was grouped into ‘living alone’ and ‘living with a partner’ (married or registered partnership).

Anaemia severity was categorised according to the definitions by the World Health Organization into ‘mild’ (110 g/L-normal value), ‘moderate’ (80–110 g/L), and ‘severe’ (<80 g/L) [19]. Information on comorbidity was obtained from the Psychiatric Central Research Register (PCRR) and the National Patient Register (NPR) [11]. Comorbidity was registered for the ten years preceding the index date. It included 11 chronic disease groups: cardiovascular diseases, hypertension, chronic mental illness, diabetes, chronic obstructive pulmonary disease, chronic neurological disorders, chronic arthritis, chronic bowel disease, chronic liver disease, chronic kidney disease, and cancer (Supplementary File 1) [20,21]. The number of comorbidities was categorised into 0, 1, 2, and ≥3.

### Statistical analysis

The data were described as frequencies and proportions expressed as percentages and 95% confidence intervals (CI). We calculated adjusted proportions based on predictions at age 70–79 years, following multinomial logistic regression analysis in the case of anaemia classification and logistic regression analysis in the case of subsequent laboratory tests. The adjusted proportions were stratified by sex and presented as percentages. To investigate the possible associations between patient characteristics and having unclassified anaemia, we estimated the odds ratios (ORs) by applying logistic regression analysis. We performed a test for linear trends to assess dose-response effects. Analyses were stratified by sex and adjusted for age group. We calculated adjusted proportions of patients with unclassified anaemia according to patient characteristics. Adjusted proportions were based on predictions at age 70–79 years, following logistic regression analysis, stratified by sex and presented as percentages. Standard errors in all analyses were modelled to allow for intragroup correlations due to clusters of patients within general practice. All analyses were performed with Stata^®^ version 15.

## Results

Of the 62,731 included patients, 35,075 (55.9%) were men (Table 1). Among men, the mean age was 69.3 years, 93.3% had mild anaemia, and 53.2% had at least one comorbidity. Among women, the mean age was 65.1 years, 60.4% had mild anaemia, and 47.0% had at least one comorbidity.

**Table 1.** Demographic and clinical characteristics of individuals aged 40–90 years with new-onset anaemia detected in general practice (*n* = 62,731).

Patient characteristics	Men, n (%)	Women, n (%)	Total, n (%)
Total^a^	35,075 (55.9)	27,656 (44.1)	62,731 (100.0)
Age groups, years			
40–49	2,567 (7.3)	7,256 (26.2)	9,823 (15.7)
50–59	4,990 (14.2)	3,882 (14.0)	8,872 (14.1)
60–69	8,848 (25.2)	3,714 (13.4)	12,562 (20.0)
70–79	11,265 (32.1)	6,012 (21.7)	17,277 (27.5)
80–89	7,405 (21.1)	6,792 (24.6)	14,197 (22.6)
Educational level			
Low	13,618 (38.8)	13,312 (48.1)	26,930 (42.9)
Medium	15,193 (43.3)	8,798 (31.8)	23,991 (38.2)
High	6,264 (17.9)	5,546 (20.1)	11,810 (18.8)
Income			
Low	11,853 (33.8)	9,045 (32.7)	20,898 (33.3)
Medium	10,709 (30.5)	10,159 (36.7)	20,868 (33.3)
High	12,513 (35.7)	8,452 (30.6)	20,965 (33.4)
Civil status			
Living with a partner	21,834 (62.2)	13,464 (48.7)	35,298 (56.3)
Living alone	13,241 (37.8)	14,192 (51.3)	27,433 (43.7)
Anaemia severity^b^			
Mild	32,736 (93.3)	16,693 (60.4)	49,429 (78.8)
Moderate	1,999 (5.7)	10,047 (36.3)	12,046 (19.2)
Severe	340 (1.0)	916 (3.3)	1,256 (2.0)
Number of comorbidities			
0	16,457 (46.9)	14,654 (53.0)	31,111 (49.6)
1	9,180 (26.2)	6,421 (23.2)	15,601 (24.9)
2	5,876 (16.8)	3,943 (14.3)	9,819 (15.7)
≥3	3,562 (10.2)	2,638 (9.5)	6,200 (9.9)
Type of comorbidity^c^			
Cardiovascular disease	10,577 (30.2)	5,672 (20.5)	16,249 (25.9)
Hypertension	7,890 (22.5)	6,179 (22.3)	14,069 (22.4)
Mental illness	2,395 (6.8)	3,012 (10.9)	5,407 (8.6)
Diabetes	3,835 (10.9)	2,591 (9.4)	6,426 (10.2)
Chronic obstructive pulmonary disease	2,158 (6.2)	1,657 (6.0)	3,815 (6.1)
Neurological disorder	1,141 (3.3)	743 (2.7)	1,884 (3.0)
Arthritis	226 (0.6)	401 (1.4)	627 (1.0)
Inflammatory bowel disease	317 (0.9)	302 (1.1)	619 (1.0)
Liver disease	480 (1.4)	333 (1.2)	813 (1.3)
Kidney disease	622 (1.8)	422 (1.5)	1,044 (1.7)
Cancer	3,041 (8.7)	1,801 (6.5)	4,842 (7.7)

^a^Total numbers are shown in row percentages; all other variables are shown in column percentages.

^b^Anaemia severity was defined according to WHO’s guidelines: mild anaemia (110 g/L-normal value), moderate anaemia (80–110 g/L), and severe anaemia (<80 g/L).

^c^A person was categorised as having comorbidity within the specified chronic disease group (e.g. cardiovascular disease).

If the person had any of the included diseases (e.g. ischaemic heart disease) listed under the chronic disease group (Supplementary File 1).

### Anaemia classification

The aetiology of anaemia could not be determined according to the algorithm in 78.9% (CI: 77.3–80.5) of men and 65.1% (CI: 63.4–66.9) of women due to missing blood tests.

The morphology of the erythrocyte could not be determined in 45.8% (CI: 43.0–48.6) of men and 39.6% (CI: 37.2–42.0) of women as the MCV blood test was not requested.

**Figure 1.**
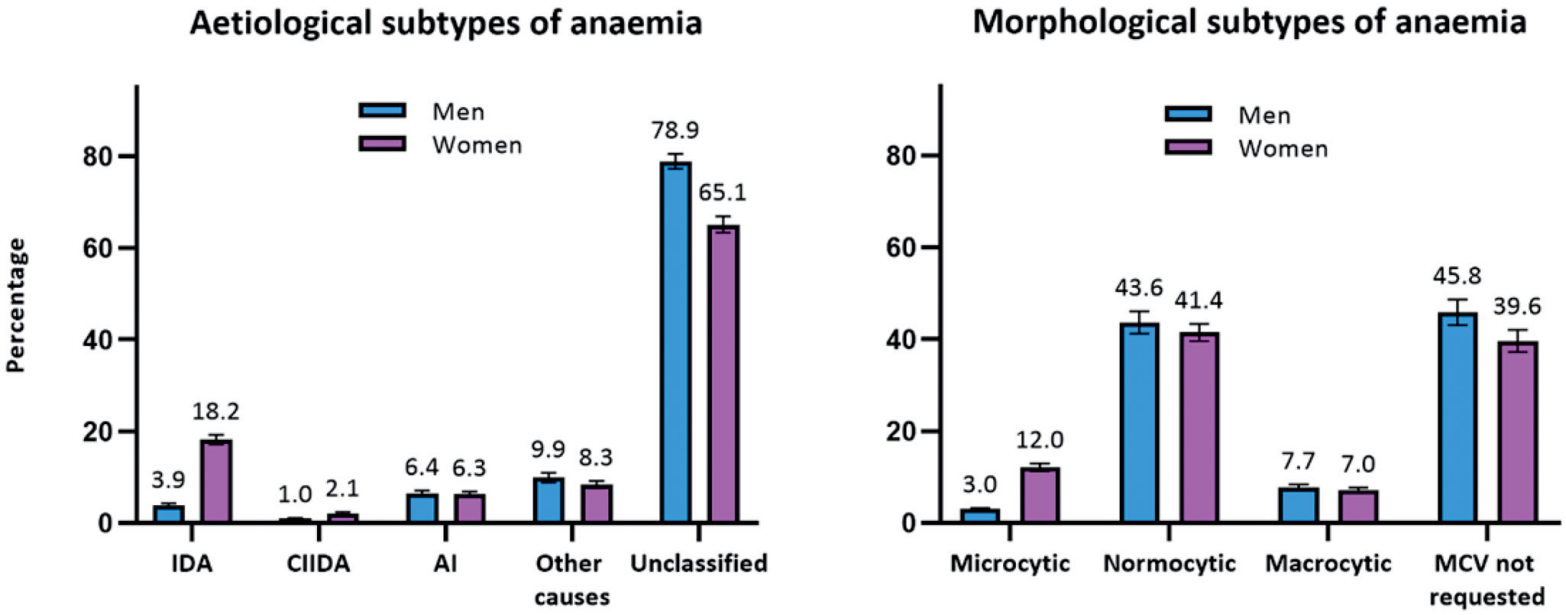
Percentage^a^ of patients with anaemia classified into subtypes of anaemia (*n* = 62,731). AI: anaemia of inflammation; CIIDA: combined inflammatory iron deficiency anaemia; IDA: iron deficiency anaemia; MCV: mean cell volume. ^a^Adjusted percentages were calculated by setting age at 70–79 years. Error bars = 95% confidence intervals.

The classification into anaemia subtypes is displayed in Figure 1.

### Laboratory tests

**Figure 2.**
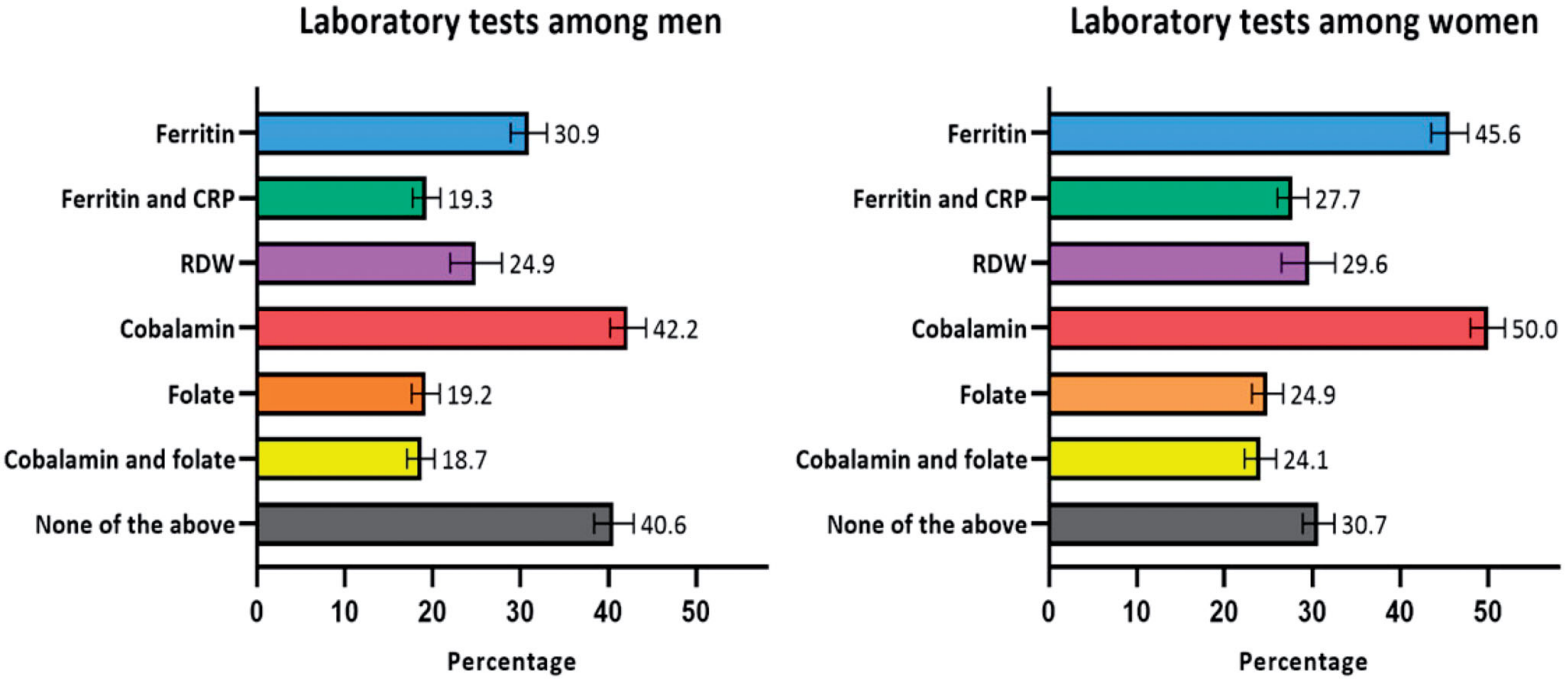
Percentage^a^ of patients with anaemia who received subsequent laboratory tests in the following 3 months (*n* = 62,731). CRP: C-reactive protein; RDW: red cell distribution width. ^a^Adjusted percentages were calculated by setting age at 70–79 years. Error bars = 95% confidence intervals.

The percentages of patients with subsequent laboratory tests are displayed in Figure 2. In 40.6% (CI: 38.4–42.9) of men and 30.7% (CI: 28.9–32.5) of women, none of the included laboratory tests were performed. Moreover, in the group with unclassified anaemia, 50.8% (CI: 48.1-53.5) of men and 48.6% (CI: 46.0-51.3) of women had none of the additional laboratory tests performed. In 30.3% (CI: 29.0–31.6) of men and 21.6% (CI: 20.5–22.8) of women, the haemoglobin analyses were not repeated within 12 months. The ‘laboratory test package’ was used in 9.5% (CI: 8.2–10.2) of men and 13.4% (CI: 11.6–15.1) of women in the Central Denmark Region (23,316 men, 18,728 women).

The percentages of patients with subsequent laboratory tests (stratified by morphological subtypes) are displayed in Supplementary Table 1.

### Associations between unclassified anaemia and patient characteristics

The likelihood and percentage of patients having unclassified anaemia according to the algorithm and the association with specific patient characteristics are displayed in Table 2. Increasing age was associated with unclassified anaemia in both sexes in a dose-response manner (test for linear trend: *p* < 0.001). The highest likelihood was seen in patients aged 80–89 years compared to patients aged 40–49 years with an OR 1.86 (CI: 1.68–2.07) in men and OR 3.00 (CI: 2.73–3.29) in women.

**Table 2.** The likelihood and percentage of patients having unclassified anaemia according to the aetiological algorithm and the association with patient characteristics (*n* = 62,731).

Patient characteristics	Men		Women	
	OR (CI)^a^	Percentage (CI)^b^	OR (CI)^a^	Percentage (CI)^b^
Age groups, years				
40–49	1	69.3% (66.9–71.7)	1	43.2% (41.1–45.4)
50–59	1.25 (1.13–1.40)	73.9% (71.8–76.0)	1.48 (1.36–1.61)	53.0% (50.9–55.2)
60–69	1.45 (1.31–1.61)	76.6% (74.9–78.4)	1.96 (1.78–2.15)	59.8% (57.7–62.0)
70–79	1.68 (1.52–1.86)	79.1% (77.5–80.8)	2.40 (2.20–2.62)	64.7% (62.7–66.6)
80–89	1.86 (1.68–2.07)	80.8% (79.1–82.4)	3.00 (2.73–3.29)	69.5% (67.6–71.5)
Educational level				
Low	1	79.4% (77.7–81.2)	1	65.3% (63.4–67.2)
Medium	1.01 (0.95–1.08)	79.6% (78.0–81.3)	0.89 (0.84–0.95)	62.7% (60.7–64.6)
High	0.99 (0.91–1.09)	79.3% (77.4–81.2)	0.81 (0.76–0.87)	60.5% (58.3–62.6)
Income				
Low	1	78.7% (77.0–80.4)	1	64.2% (62.2–66.2)
Medium	1.07 (1.01–1.15)	79.9% (78.3–81.5)	1.05 (0.99–1.12)	65.4% (63.5–67.2)
High	1.08 (1.01–1.16)	79.9% (78.2–81.6)	0.84 (0.78–0.90)	60.2% (58.1–62.3)
Civil status				
Living alone	1	79.7% (78.1–81.4)	1	64.8% (62.9–66.6)
Living with a partner	0.99 (0.94–1.05)	79.6% (77.9–81.2)	0.91 (0.87–0.96)	62.7% (60.8–64.6)
Anaemia severity^b^				
Mild	1	81.4% (79.8–82.9)	1	71.8% (70.0–73.5)
Moderate	0.25 (0.23–0.28)	52.5% (49.8–55.2)	0.40 (0.38–0.43)	50.6% (48.6–52.7)
Severe	0.21 (0.17–0.27)	48.1% (42.2–53.9)	0.24 (0.20–0.28)	37.6% (33.8–44.4)
No. of comorbidities				
0	1	78.2% (76.5–80.0)	1	59.2% (57.2–61.1)
1	1.08 (1.02–1.15)	79.6% (77.8–81.3)	1.38 (1.30–1.47)	66.7% (64.7–68.7)
2	1.14 (1.05–1.23)	80.4% (78.6–82.2)	1.44 (1.33–1.55)	67.6% (65.4–69.7)
≥3	1.25 (1.14–1.37)	81.8% (80.0–83.6)	1.44 (1.31–1.58)	67.6% (65.3–69.9)
Type of comorbidity^c^				
Cardiovascular disease	1.08 (1.02–1.14)	80.4% (78.7–82.0)	1.13 (1.06–1.21)	65.8% (63.8–67.9)
Hypertension	1.12 (1.05–1.19)	80.9% (79.3–82.5)	1.23 (1.15–1.31)	67.0% (65.0–69.1)
Mental illness	1.08 (0.98–1.20)	80.8% (78.7–82.9)	1.28 (1.18–1.39)	68.8% (66.6–71.0)
Diabetes	1.34 (1.21–1.47)	83.5% (81.8–85.1)	1.27 (1.16–1.39)	68.4% (66.1–70.7)
COPD	0.92 (0.82–1.03)	78.4% (76.0–80.7)	1.09 (0.98–1.21)	65.5% (62.8–68.2)
Neurological disorder	1.12 (0.96–1.29)	81.3% (78.8–83.8)	1.30 (1.11–1.52)	69.3% (65.8–72.9)
Arthritis	1.02 (0.75–1.40)	80.0% (74.6–85.4)	1.38 (1.10–1.74)	70.7% (65.6–75.8)
IBD	1.02 (0.79–1.31)	79.9% (75.6–84.2)	1.22 (0.97–1.52)	68.1% (62.9–73.2)
Liver disease	0.92 (0.75–1.13)	78.3% (74.4–82.2)	1.13 (0.90–1.42)	66.4% (61.2–71.7)
Kidney disease	1.22 (0.99–1.50)	82.6% (79.4–85.8)	1.50 (1.21–1.86)	72.3% (67.7–76.9)
Cancer	1.04 (0.95–1.15)	80.2% (78.2–82.2)	1.39 (1.25–1.54)	70.3% (67.7–72.9)

CI: 95% confidence intervals; COPD: chronic obstructive pulmonary disease; IBD: inflammatory bowel disease; OR: odds ratio.

^a^Odds ratios (of having unclassified anaemia *vs.* classified anaemia according to patient characteristics) were adjusted for age.

^b^Adjusted percentages were calculated by setting the age at 70–79 years.

^c^Anaemia severity was defined according to WHO’s guidelines: mild anaemia (110 g/L-normal value), moderate anaemia (80–110 g/L), and severe anaemia (<80 g/L).

^d^A person was categorised as having comorbidity within the specified chronic disease group (e.g. cardiovascular disease) if the person had any of the included diseases (e.g. ischaemic heart disease) listed under the chronic disease group (Supplementary File 1).

^e^The reference group for each of the comorbidities is patients without the specified comorbidity (e.g. the reference group for cardiovascular disease in patients without cardiovascular disease).

Women with high educational levels were less likely to have unclassified anaemia compared to women with low educational levels (OR: 0.84, CI: 0.78–0.90). Men with high income (OR: 1.08, CI: 1.01–1.16) were more likely to have unclassified anaemia compared to men with low income. Women living with a partner (OR: 0.91, CI: 0.87–0.96) were less likely to have unclassified anaemia compared to women living alone.

Increasing severity of anaemia was associated with a lower risk of unclassified anaemia in a dose-response manner for both sexes (test for linear trend: *p* < 0.001) (Table 2). In patients with severe anaemia, the likelihood of unclassified anaemia was generally low in both men (OR: 0.21, CI: 0.17–0.27) and women (OR: 0.24, CI: 0.20–0.28). Severe anaemia was unclassified in 48.1% of men and 37.6% of women.

For both sexes, comorbidity was associated with having unclassified anaemia in a dose-response manner (test for linear trend: *p* < 0.001); the highest OR was seen in men with diabetes (OR: 1.34, CI: 1.21–1.47) and in women with chronic kidney disease (OR: 1.50, CI: 1.21–1.86).

## Discussion

### Principal findings

This population-based study including more than 62,000 patients revealed that seven to eight out of ten patients had unclassified anaemia. This suggests inadequate investigation to identify the underlying cause. For both sexes, the likelihood of unclassified anaemia increased with increasing age and increasing comorbidity. Moreover, for both sexes, severe anaemia increased the likelihood of classified anaemia. Nevertheless, half of the men and one-third of the women with severe anaemia were unclassified. A small minority of the patients with anaemia (residing in the Central Denmark Region) received the anaemia ‘laboratory test package’.

### Strengths and limitations

This study has several strengths, including the large dataset on patients with free access to the Danish healthcare system [12]. The registries hold valid data of high completeness, which ensures complete follow-up [12,22]. This limited the risk of both selection and information bias due to missing data. The high number of included patients made stratification possible and ensured high statistical precision. Additionally, the laboratory information systems comply with international standards and relevant quality criteria. The direct recording of all data into the laboratory information systems reduced the risk of data loss [10]. The high extent of similarity in healthcare usage across the five regions suggests that our results are generalisable to the rest of Denmark [23]. The findings may also be generalised to similar healthcare systems based on GP gatekeeping.

A potential limitation is that point-of-care tests (POCTs) performed by GPs are not recorded in the laboratory information systems. To classify anaemia into different subtypes, blood tests must be analysed at hospital laboratories. Hence, if a GP did not follow up on POCT-detected anaemia, our estimates of unclassified anaemia may have been underestimated, and our estimates of new-onset anaemia may have been overestimated. Equivalently, a repeated haemoglobin sample may also be measured by POCTs, and this holds a potential risk of underestimating the degree of follow-up.

No standard definition of ‘new-onset anaemia’ exists. Various definitions have been used, including no anaemia in the previous 12 months [24] or 24 months [25]. We defined it as no anaemia in the previous 15 months (detected in general practice or at a hospital), excluding, e.g. comorbid patients with chronic anaemia.

Laboratory tests are commonly performed in general practice, and their use has increased over time [26]. However, we had no information on the indications for requesting the laboratory tests. Further, information on referrals to, e.g. cancer patient pathways or acute admissions was not included in this study.

### Findings in relation to other studies

We identified no other studies exploring whether patients with new-onset anaemia in general practice can be classified into different subtypes based on subsequent blood tests. Thus, our findings are currently unchallenged and should be reassessed in other studies.

Although complex algorithms for classifying anaemia based on aetiology exist, we used a simple algorithm [7,16], which was clinically applicable and used previously [7,27]. This algorithm includes the two most common aetiological subtypes: iron deficiency anaemia and anaemia of inflammation [28]. Furthermore, the algorithm includes ferritin in combination with CRP. As ferritin is an acute phase reactant, it may be elevated under inflammatory conditions [6]. Thus, ferritin should be interpreted in conjunction with CRP [6,7].

The few existing studies evaluating anaemia in general practice were flawed by including only an elderly population [9], using a small sample size [8], and reporting no inclusion criteria for new-onset anaemia [8,9]. Nevertheless, in line with our findings, they reported that most patients had insufficient laboratory testing performed and that patients with severe anaemia were more likely to undergo further laboratory testing [8,9].

We found a large amount of mild anaemia in men (93.3%) compared to women (60.4%). This may be due to the differences in haemoglobin threshold for defining anaemia in men and women [15], as the thresholds for mild, moderate and severe anaemia do not differ across the sexes [19].

We identified no other studies exploring whether unclassified anaemia is associated with sociodemographic and clinical factors (except for anaemia severity). However, a previous study revealed that the distribution of diagnostic delay is affected by social inequality, specifically low socioeconomic status, low educational level, and living alone [29]. Likewise, our results revealed that women with a high educational level and women living with a partner less often had unclassified anaemia compared to women with a low educational level and women living alone. In contrast, men with a high income more often had unclassified anaemia compared to men with a low income. The reasons for this finding are unclear and warrant further investigation.

The increasing association between increasing age and unclassified anaemia is noteworthy. The risk of a serious cause of anaemia, such as cancer, increases with age. Thus, this paradox might lead to less investigation among those with the highest risk of developing a serious disease, such as cancer.

### Meaning of the study

Aetiological classification of anaemia is essential to ensure relevant investigation and timely diagnosis of potentially severe disease [6,7]. Our results indicate that the majority of new-onset anaemia was not classified, although such classification could have served as important information in the further diagnostic process. When a haemoglobin test is requested, compulsory use of the ‘laboratory test package’ could ensure that several relevant blood parameters are investigated. Such automated laboratory testing is also likely to reduce the number of GP visits and diagnostic delays [30].

Our results illustrate the importance of careful evaluation of patients with anaemia. Future interventions are called for to increase the awareness of patients with anaemia and to ensure adequate laboratory testing and follow-up of these patients. Additional research is required to examine the diagnostic workup of patients with anaemia (e.g. referrals in cancer diagnostic pathways) and to determine the clinical outcomes of patients with unclassified anaemia.

## Supplementary Material

Supplementary_file_I.docx

Supplementary_table_I.docx
