# Supplementary material for: Insufficient classification of anaemia in general practice: a Danish register-based observational study
Source: Scand J Prim Health Care. 2021 Jul 30;39(3):364–72. doi: 10.1080/02813432.2021.1958499 (PMC8475152; doi:10.1080/02813432.2021.1958499)
Supplement: Supplementary_file_I.docx [file IPRI_A_1958499_SM3605.docx]

**Supplementary file 1. Chronic disease groups (CDGs) and diseases included with the corresponding ICD-10 codes (International Classification of Diseases,** 10^th^ revision**)**

| **Chronic disease groups** | **Diseases** | **ICD-10 codes** |
| --- | --- | --- |
| **Cardiovascular disease** | Cardiac valve diseases | I08, I09, I38, I39, Z95 |
|  | Angina pectoris | I20 |
|  | Acute myocardial infarction | I21, I22 |
|  | Ischaemic heart disease | I24-I25 |
|  | Atrial fibrillation | I48 |
|  | Heart failure | I50, I11.0, I13.0, I13.2 |
|  | Apoplexy | I60-I69, G45, G46 |
| **Hypertension** | Hypertension | I10-I15 |
| **Mental illness** | Dementia | F00-F03, F05.1, G30 |
|  | Schizophrenia | F20 |
|  | Psychotic disorders | F22-F25, F28-F29 |
|  | Affective disorders | F30-39 |
|  | Anxiety | F40-F41 |
|  | Eating disorder | F50.0, F50.2 |
| **Diabetes** | Diabetes | E10-E14 |
| **Chronic obstructive** | Chronic obstructive pulmonary disease | DJ40, DJ409, DJ41, DJ410, DJ411, |
| **pulmonary disease** |  | DJ418, DJ42, DJ429, DJ429A, DJ429B, |
|  |  | DJ43, DJ430, DJ430A, DJ431, DJ431A, DJ432, DJ438, DJ439, DJ439A, DJ44, DJ440, DJ441, DJ448, DJ448A, DJ448B, DJ449, DJ47, DJ479, DJ96, DJ960, DJ961, DJ969 |
| **Neurological disorder** | Parkinson’s disease | G20-G22 |
|  | Multiple sclerosis | G35 |
|  | Epilepsy | G40 (excl. G40.4), G41 |
| **Arthritis** | Rheumatoid arthritis | M05, M06, M790 |
| **Inflammatory bowel disease** | Crohn’s disease | K50 |
|  | Ulcerative colitis | K51 |
| **Liver disease** | Viral hepatitis | B18 |
|  | Liver disease | K70, K71.3-K71.5, K71.7, K72.1, |
|  |  | K72.7, K72.9, K73-K74, K76 |
| **Kidney disease** | Kidney disease | N18+N19 |
| **Cancer** | Cancer excl. non-melanoma skin cancer | C00-C96 (excl. C44) |
