# Supplementary material for: Insufficient classification of anaemia in general practice: a Danish register-based observational study
Source: Scand J Prim Health Care. 2021 Jul 30;39(3):364–72. doi: 10.1080/02813432.2021.1958499 (PMC8475152; doi:10.1080/02813432.2021.1958499)
Supplement: Supplementary_table_I.docx [file IPRI_A_1958499_SM3604.docx]

**Supplementary table I.** Percentage of patients with anaemia who received subsequent laboratory tests stratified by morphological subtypes of anaemia (n=62,731)

| **Laboratory tests** | **Microcytic anaemia**  Percentage (CI) | **Normocytic anaemia**  Percentage (CI) | **Macrocytic anaemia**  Percentage (CI) | **MCV not requested**  Percentage (CI) |
| --- | --- | --- | --- | --- |
| **Ferritin** |  |  |  |  |
| Men | 67.0 (63.6-70.4) | 44.7 (41.5-48.0) | 47.0 (43.3-50.6) | 13.3 (11.3-15.4) |
| Women | 69.1 (66.5-71.8) | 59.4 (56.3-62.4) | 57.2 (53.6-60.7) | 20.7 (18.5-23.0) |
| **Ferritin and CRP** |  |  |  |  |
| Men | 39.6 (36.2-43.0) | 28.7 (26.0-31.4) | 28.9 (25.8-32.0) | 7.7 (6.3-9.2) |
| Women | 39.8 (37.1-42.6) | 37.9 (35.2-40.7) | 37.0 (33.4-40.5) | 11.1 (9.5-12.7) |
| **RDW** |  |  |  |  |
| Men | 55.8 (50.9-60.7) | 45.5 (40.3-50.7) | 48.8 (43.4-54.3) | 0.2 (0.2-0.3) |
| Women | 54.9 (50.4-59.4) | 45.8 (40.8-50.7) | 46.5 (41.3-51.8) | 0.3 (0.1-0.5) |
| **Cobalamin** |  |  |  |  |
| Men | 61.0 (57.6-64.4) | 52.1 (49.2-55.0) | 60.7 (57.7-63.8) | 28.9 (26.3-31.5) |
| Women | 63.8 (61.3-66.3) | 57.0 (54.4-59.6) | 61.9 (58.7-65.1) | 35.1 (32.6-37.7) |
| **Folate** |  |  |  |  |
| Men | 36.5 (32.6-40.5) | 28.5 (25.9-31.1) | 37.9 (34.7-41.1) | 6.5 (5.2-7.7) |
| Women | 37.6 (34.6-40.7) | 32.2 (29.6-34.8) | 38.4 (35.2-41.6) | 10.1 (8.7-11.5) |
| **Cobalamin and folate** |  |  |  |  |
| Men | 35.6 (31.7-39.5) | 27.7 (25.1-30.3) | 36.9 (33.7-40.1) | 6.2 (5.0-7.4) |
| Women | 36.6 (33.6-39.6) | 31.2 (28.7-33.8) | 36.7 (33.5-39.9) | 9.6 (8.3-11.0) |
| **None of the above** |  |  |  |  |
| Men | 10.2 (8.1-12.2) | 17.6 (14.9-20.3) | 12.8 (10.4-15.1) | 68.4 (65.7-71.1) |
| Women | 7.9 (6.5-9.3) | 13.7 (11.4-15.9) | 11.4 (9.2-13.6) | 60.7 (57.9-63.4) |

Abbreviations: CI: 95% confidence interval, CRP: C-reactive protein, MCV: mean cell volume, RDW: red cell distribution width.

^a^ Adjusted percentages were calculated by setting age at 70-79 years.
